# Supplementary material for: The causal relationship between the human gut microbiota and pyogenic arthritis: a Mendelian randomization study
Source: Front Cell Infect Microbiol. 2024 Nov 26;14:1452480. doi: 10.3389/fcimb.2024.1452480 (PMC11629706; doi:10.3389/fcimb.2024.1452480)
Supplement: Supplementary file 1 [file DataSheet1.zip › mendelian test/ebi-a-GCST90027703.csv_leave_one_out.pdf]

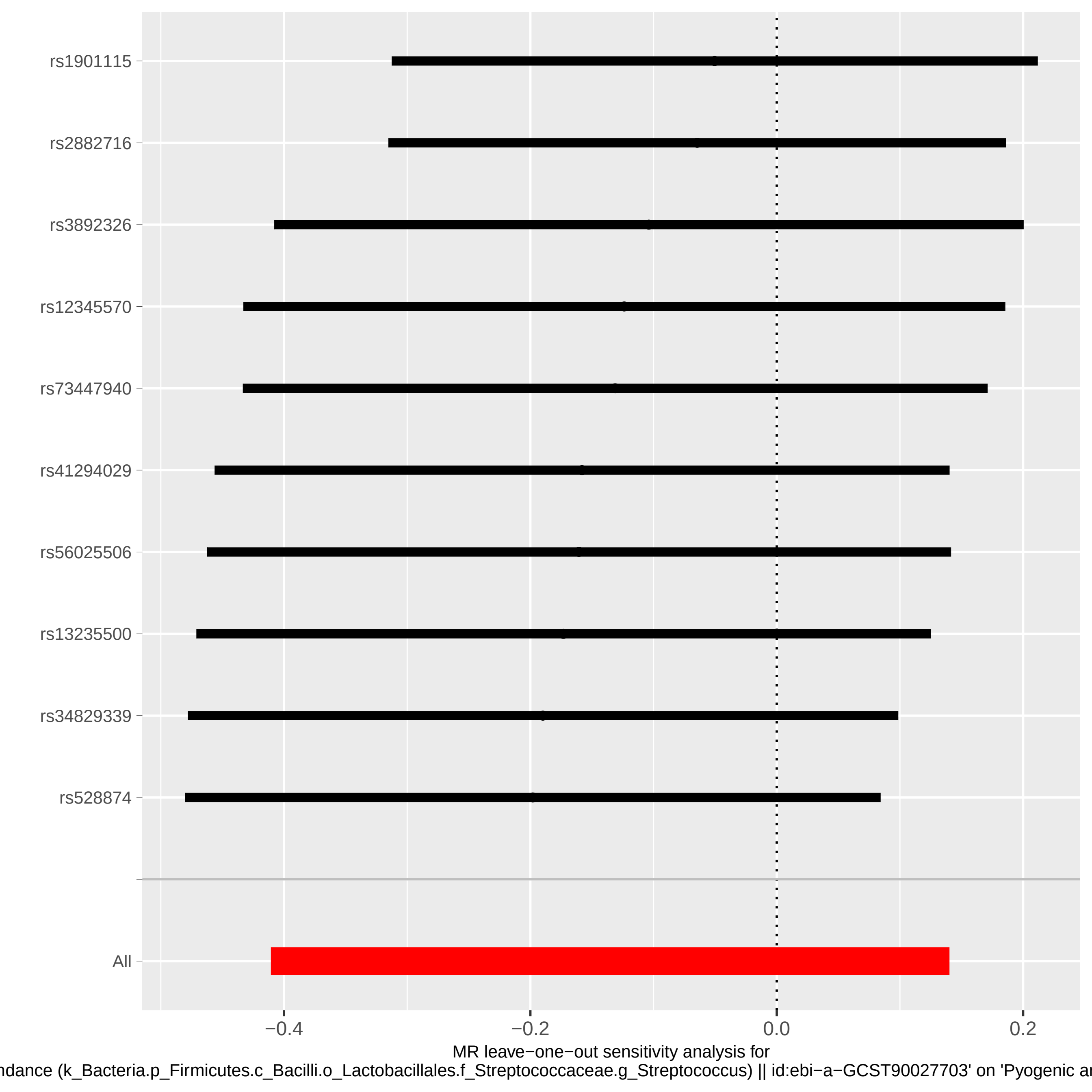

rs1901115
rs2882716
rs3892326
rs12345570
rs73447940
rs41294029
rs56025506
rs13235500
rs34829339
rs528874
All
−0.4
−0.2
0.0
0.2
MR leave−one−out sensitivity analysis for
ndance (k_Bacteria.p_Firmicutes.c_Bacilli.o_Lactobacillales.f_Streptococcaceae.g_Streptococcus) || id:ebi−a−GCST90027703' on 'Pyogenic ar
